# *In Situ* Studies of Arylboronic Acids/Esters and R_3_SiCF_3_ Reagents: Kinetics, Speciation, and Dysfunction at the Carbanion–Ate Interface

**DOI:** 10.1021/acs.accounts.2c00113

**Published:** 2022-04-18

**Authors:** Andrés García-Domínguez, Andrew G. Leach, Guy C. Lloyd-Jones

**Affiliations:** † EaStChem, University of Edinburgh, Joseph Black Building, David Brewster Road, Edinburgh EH9 3FJ, U.K.; ‡ School of Health Sciences, Stopford Building, The University of Manchester, Oxford Road, Manchester M13 9PT, U.K.

## Abstract

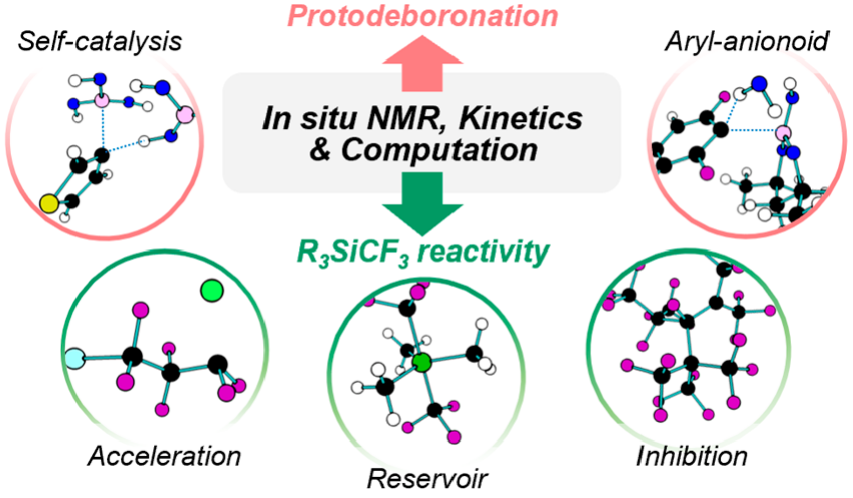

Reagent instability reduces the efficiency of chemical processes, and while much effort is devoted to reaction optimization, less attention is paid to the mechanistic causes of reagent decomposition. Indeed, the response is often to simply use an excess of the reagent. Two reaction classes with ubiquitous examples of this are the Suzuki–Miyaura cross-coupling of boronic acids/esters and the transfer of CF_3_ or CF_2_ from the Ruppert–Prakash reagent, TMSCF_3_. This Account describes some of the overarching features of our mechanistic investigations into their decomposition. In the first section we summarize how specific examples of (hetero)arylboronic acids can decompose via aqueous protodeboronation processes: Ar–B(OH)_2_ + H_2_O → ArH + B(OH)_3_. Key to the analysis was the development of a kinetic model in which pH controls boron speciation and heterocycle protonation states. This method revealed six different protodeboronation pathways, including self-catalysis when the pH is close to the p*K*_a_ of the boronic acid, and protodeboronation via a transient aryl anionoid pathway for highly electron-deficient arenes. The degree of “protection” of boronic acids by diol-esterification is shown to be very dependent on the diol identity, with six-membered ring esters resulting in faster protodeboronation than the parent boronic acid. In the second section of the Account we describe ^19^F NMR spectroscopic analysis of the kinetics of the reaction of TMSCF_3_ with ketones, fluoroarenes, and alkenes. Processes initiated by substoichiometric “TBAT” ([Ph_3_SiF_2_][Bu_4_N]) involve anionic chain reactions in which low concentrations of [CF_3_]^−^ are rapidly and reversibly liberated from a siliconate reservoir, [TMS(CF_3_)_2_][Bu_4_N]. Increased TMSCF_3_ concentrations reduce the [CF_3_]^−^ concentration and thus inhibit the rates of CF_3_ transfer. Computation and kinetics reveal that the TMSCF_3_ intermolecularly abstracts fluoride from [CF_3_]^−^ to generate the CF_2_, in what would otherwise be an endergonic α-fluoride elimination. Starting from [CF_3_]^−^ and CF_2_, a cascade involving perfluoroalkene homologation results in the generation of a hindered perfluorocarbanion, [C_11_F_23_]^−^, and inhibition. The generation of CF_2_ from TMSCF_3_ is much more efficiently mediated by NaI, and in contrast to TBAT, the process undergoes autoacceleration. The process involves NaI-mediated α-fluoride elimination from [CF_3_][Na] to generate CF_2_ and a [NaI·NaF] chain carrier. Chain-branching, by [(CF_2_)_3_I][Na] generated *in situ* (CF_2_ + TFE + NaI), causes autoacceleration. Alkenes that efficiently capture CF_2_ attenuate the chain-branching, suppress autoacceleration, and lead to less rapid difluorocyclopropanation. The Account also highlights how a collaborative approach to experiment and computation enables mechanistic insight for control of processes.

## Key References

- CoxP. A.; LeachA. G.; CampbellA. D.; Lloyd-JonesG. C.Protodeboronation of Heteroaromatic, Vinyl, and Cyclopropyl Boronic Acids: pH-Rate Profiles, Autocatalysis, and DisproportionationJ. Am. Chem. Soc.2016, 138, 9145–91572735597310.1021/jacs.6b03283.^1^ Development of a speciation-kinetics model to account for empirical pH–log *k*_obs_ profiles in protodeboronation of heteroaromatic boronic acids.
- HayesH. L. D.; WeiR.; AssanteM.; GeogheghanK. J.; JinN.; TomasiS.; NoonanG.; LeachA. G.; Lloyd-JonesG. C.Protodeboronation of (Hetero)Arylboronic Esters: Direct versus Prehydrolytic Pathways and Self-/Auto-Catalysis. J. Am. Chem. Soc.2021, 143, 14814–148263446023510.1021/jacs.1c06863.^2^ A rationalization of the protolytic instability of boronic esters, under aqueous basic conditions, using stopped-flow NMR and computation.
- JohnstonC. P.; WestT. H.; DooleyR. E.; ReidM.; JonesA. B.; KingE. J.; LeachA. G.; Lloyd-JonesG. C.Anion-Initiated Trifluoromethylation by TMSCF_3_: Deconvolution of the Siliconate-Carbanion Dichotomy by Stopped-Flow NMR/IR. J. Am. Chem. Soc.2018, 140, 11112–111243008097310.1021/jacs.8b06777PMC6133236.^3^ Detailed kinetic analysis of the anion-initiated reaction of TMSCF_3_with ketones, demonstrating the intermediacy of a CF_3_anionoid, and the introduction of a novel variable-ratio stopped flow NMR system.
- García-DomínguezA.; WestT. H.; PrimozicJ. J.; GrantK. M.; JohnstonC. P.; CummingG. G.; LeachA. G.; Lloyd-JonesG. C.Difluorocarbene Generation from TMSCF_3_: Kinetics and Mechanism of NaI-Mediated and Si-Induced Anionic Chain Reactions. J. Am. Chem. Soc.2020, 142, 14649–146633278680410.1021/jacs.0c06751.^4^ The use of partitioning analysis and computation to rationalize the behavior of the Ruppert–Prakash reagent under anionic initiation.

## 1 Introduction

Many organoboron and organosilicon species benefit from low toxicity, low cost, and ease of preparation, leading to numerous uses, including industrial processes.^5^ However, in some cases these reagents become unstable under the conditions of their application, leading to loss of yield or function. This Account discusses the elucidation of some of the key mechanistic features that lead to this instability in arylboronic acids/esters^6^ and in the remarkably versatile fluorochemical TMSCF_3_.^7−10^ Throughout the Account we try to highlight how strategic combinations of NMR spectroscopy,^11,12^ kinetics, byproduct analysis, pH–rate profiles, isotopes, and computation have allowed us to dissect competing reaction pathways involving organoboron and organosilicon “ate” complexes, and to explain several counterintuitive prior observations.

## 2 (Hetero)aryl Boronates

**Scheme 1:**
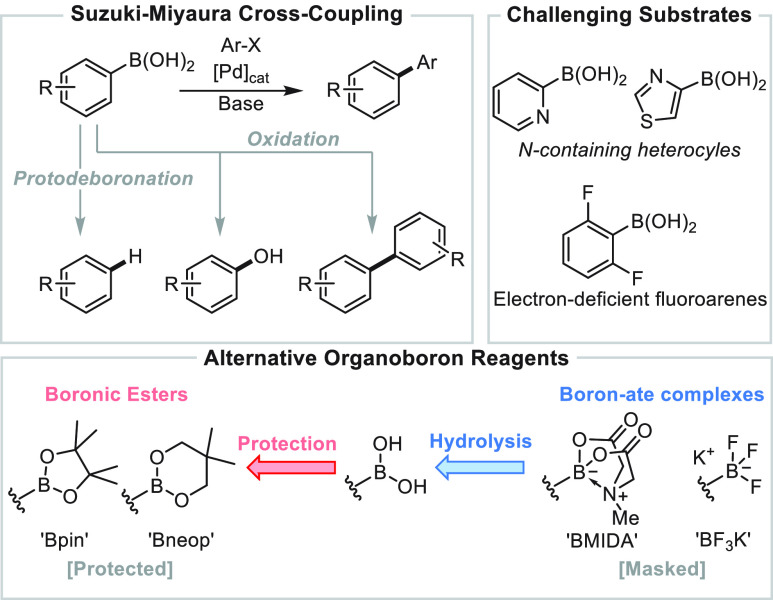
Organoboron Reagents and Suzuki–Miyaura Coupling

The Suzuki–Miyaura (SM) cross-coupling of arylboronic acids^13^ revolutionalized biaryl synthesis and remains highly valued in industry. A base is usually required to induce transfer of the aryl group from boron to the metal catalyst.^14,15^ Competing base-mediated processes (Scheme 1), including oxidation and protodeboronation, are detrimental to the efficiency.^16^ Although the oxidative processes can be minimized by careful choice of reaction conditions, the protodeboronation is mostly dependent on the identity of the boronic acid. The development of protected, or “masked”,^17^ reagents has been one of several effective strategies for mitigating the “protodeboronation problem”.^18−20^

In 2006, we began a collaborative project on ligand descriptors^21^ and used SM coupling to generate parametrization data. We encountered extensive side reactions with boronic acids and instead used ArBF_3_K reagents^22^ under Molander’s conditions.^23^ This gave substantially cleaner couplings and naturally led to a curiosity as to why this is the case.^24^ Further investigation identified that many of the beneficial effects arose from the controlled hydrolytic release of arylboronic acids *in situ*,^24a^ a process modulated by the glass surface of the reaction vessel, the stirring rate, and the pH.^24b^ With a new-found interest in “slow-release”,^17^ we collaborated with Burke, Houk, and Cheong on the base-mediated hydrolysis of BMIDA boronates.^25^*In situ* NMR, kinetics, heavy atom kinetic isotope effects (KIEs), and DFT calculations revealed two pathways, controlled by pH. One involves attack at C=O by hydroxide ion, the other B–NMe bond cleavage by neutral water.^25^

### 2.1 Protodeboronation pH–Rate Profiles

The above investigations made us appreciate that the stability of boronic acids under the conventional aqueous–organic basic conditions of SM coupling was not fully understood.^13,16^ Indeed, although mechanistic work by Kuivila^26^ in the 1960s on the protodeboronation of simple aryl boronic acids had been expanded on by Fröhn,^27^ Cammidge,^28^ Buchwald,^20^ and Perrin,^29^ reactivity trends were not readily compared, and there was scant detail on heteroaromatic boronic acids, which are systems perceived to be the most sensitive.^13,16−18^

**Figure 1:**
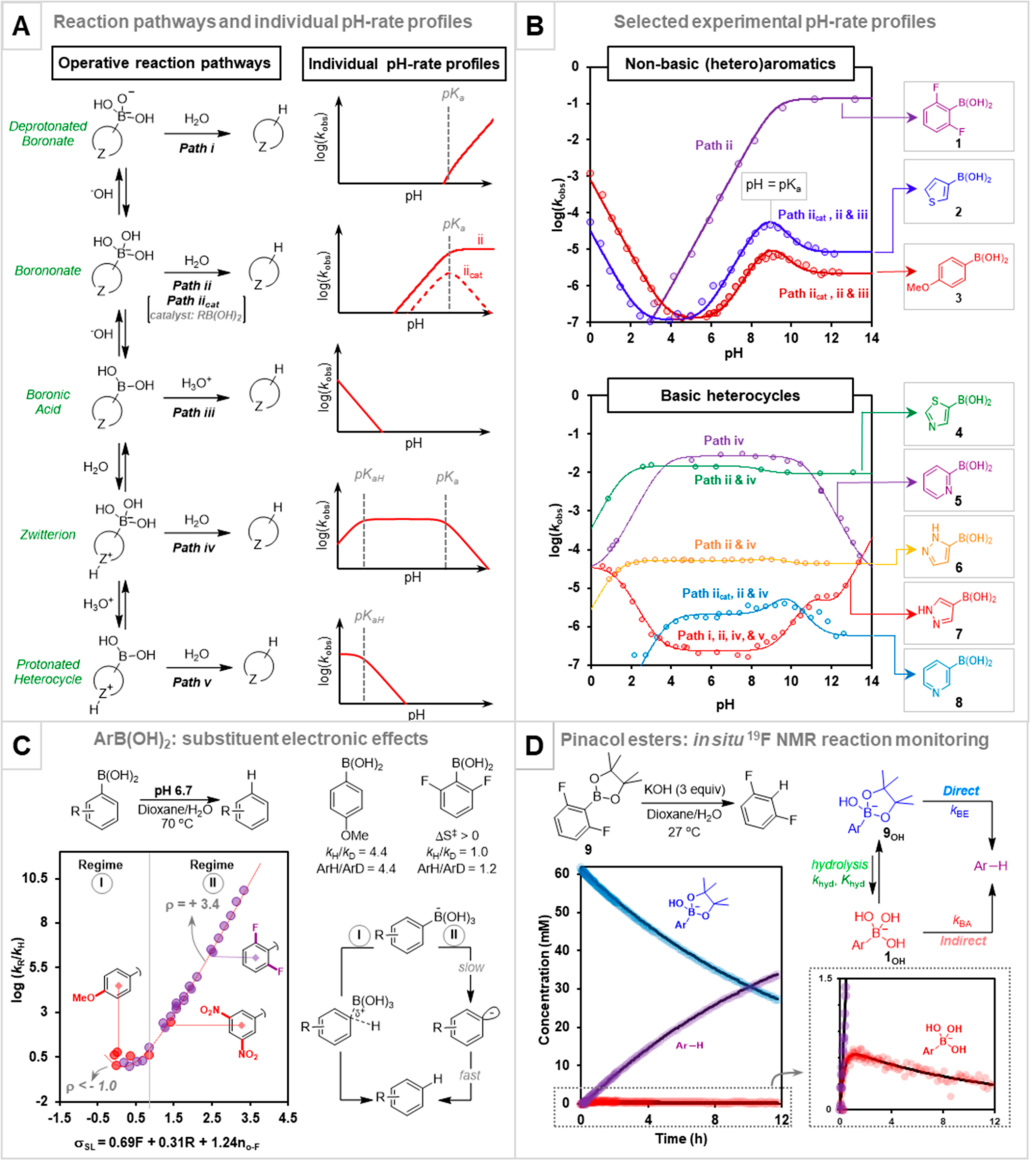
(A) Overarching kinetic model (pathways i–v) for pH-mediated speciation and protodeboronation. (B) Example pH–rate profiles for the protodeboronation of selected (hetero)arylboronic acids (50 mM, 50% vol. aq. dioxane, 70 °C). (C) Modified Swain–Lupton analysis of the protodeboronation of arylboronic acids. (D) Competing direct and indirect protodeboronation of boronate esters. Data from refs (1), (2), and (30).

A key first step was our identification that a medium of 50% aq. dioxane at 70 °C allowed the kinetics of a very wide array of boronic acids to be monitored in the presence of exogenous acids, bases, buffers, and metal salts, at concentrations amenable to NMR analysis.^11,12^ At the heart of the analysis was the nonlinear regression of pH–log *k*_obs_ profiles using a model comprising weighted combinations of six pathways (Figure 1A), where *k*_obs_ is the overall empirical pseudo-first-order rate constant.

We then analyzed the kinetics of protodeboronation of 52 different boronic acids, R–B(OH)_2_, where R is aryl, heteroaryl, (cyclo)alkyl, and vinyl.^1,30^ For 20 of these we explored the full pH scale (Figure 1B). While the impact of pH on the protodeboronation rate is, *a priori*, difficult to predict, the empirical data provide insight into the pH-controlled speciation of the boronic acid, catalysis by [H_3_O]^+^ and [OH]^−^ ions, and the identification of auto/self-catalysis, *vide infra*. This allowed categorization of the boronic acids according to specific features of the R-group, such as electron-demand, basicity, number and position of heteroatoms, and ability to coordinate metal ions.^1^

### 2.2 Nonbasic (Hetero)aromatics

Fitting the kinetic model to the pH–log *k*_obs_ profiles for simple aromatic and nonbasic heterocyclic systems required three general pathways. These proceed via a deprotonated boronate (i, Figure 1A), as suggested by Perrin;^29^ the boronate (ii); and the boronic acid (iii), the latter two pathways having been identified by Kuivila^26^ for substituted phenylboronic acids. However, for technical reasons, Kuivila’s studies were conducted at low boron concentrations and were limited to pH ≤ 6.7;^26^ in other words, under conditions very different from those commonly employed in Suzuki–Miyaura cross-couplings.^13−20^

Exploration of the basic pH region of the pH–log *k*_obs_ profiles, Figure 1B, revealed some unexpected features.^1,30^ For example, 2,6-difluorophenylboronic acid (**1**) shows a simple rise in rate to reach a plateau at a pH above the p*K*_a_, consistent with water-mediated unimolecular decomposition of the boronate (see solid line for pathway ii in Figure 1A). In contrast, the 3-thienyl (**2**), *p*-anisyl (**3**), and derivatives reach a rate maximum when the boronic/boronate speciation is equal (pH = p*K*_a_).^30^ The extent of this deviation in behavior is dependent on the initial concentration of boronic acid.

Both features were indicative that the protodeboronation of [ArB(OH)_3_]^−^ is catalyzed by ArB(OH)_2_ (Figure 1A, path ii_cat_). However, an initially confusing aspect was that overall kinetics were still first-order. This was resolved by showing, experimentally and computationally,^1^ that the process is similarly catalyzed by endogenous B(OH)_3_, *i.e.*, *k*_cat_ ≈ *k*([B(OH)_3_] + [ArB(OH)_2_]). In other words, as the protodeboronation proceeds, one catalyst is replaced by the other and pseudo-first-order kinetics are observed. Thus, conducting Suzuki–Miyaura cross-couplings at pH values close to the p*K*_a_ of the boronic acid can result in exacerbated protodeboronation.^1^ This is especially the case at high initial concentrations and another illustration of the benefits of slow-release methods which maintain a low steady-state concentration of the unstable boronate.^17^

### 2.3 Electron-Deficient Aromatics

The rapid base-mediated decomposition of 2,6-dihalogenated arylboronic acids was reported by Perrin.^29^ Their reactivity contrasted the acceleration by electron-donating *para*- and *meta*-substituents reported by Kuivila.^26^ Reassessment of the effect of aryl substituents, with a much-expanded set of 30 substrates, proved very revealing.^30^ Using Swain–Lupton parameters to weight field (*F*) and resonance (*R*), together with an empirical correction for ortho fluorine (σ_o-F_ = 1.24) gave a very asymmetric “V-shaped” plot (Figure 1C).^30^ The correlation is indicative of a change in mechanism from simple aryl rings (regime I) to very electron-deficient ones (regime II), with a significant accumulation of negative charge at the transition state in the latter.

As noted above, the pH–log *k*_obs_ profile for the 2,6-difluorophenyl system (**1**) is indicative of exclusive reaction via pathway ii, where the boronate has a half-life of about 5 s. The analogous pentafluorophenyl boronate ([C_6_F_5_B(OH)_3_]^−^) was found to have a half-life of 2.6 ms. Analysis of ^2^H, ^11^B, and ^13^C KIEs suggested rate-limiting B–C cleavage in regime II, with aryl protonation occurring after this step. Detailed computational dissection of the water networks associated with boronate fragmentation rationalized the experimental KIEs, activation entropy (Δ*S*^‡^ = +6.2 cal/molK), and substituent effects (regime II, ρ = +3.4, Figure 1C). Reinvestigation of regime I suggested concerted protonation–deboronation,^30^ rather than the stepwise S_E_Ar mechanism proposed by Kuivila.^26^

### 2.4 Basic Heterocycles

**Figure 2:**
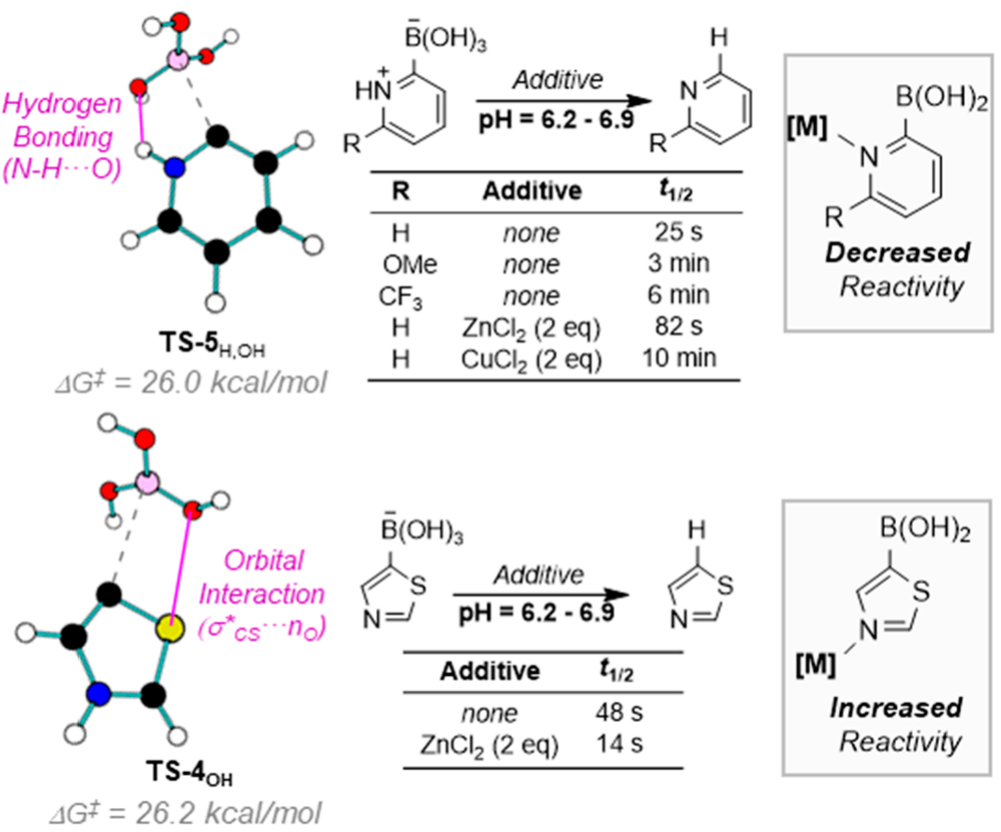
Key interactions in the protodeboronaton of 2-pyridinium (**5**_H,OH_) and 5-thiazolyl (**4**_OH_) boronates and the effects of metal-coordination. Data from ref (1).

Nonlinear regression of pH–log *k*_obs_ profiles for systems containing basic nitrogen-sites required additional pathways involving zwitterionic and cationic speciation (iv and v, Figure 1A).^1^ The studies showed the protodeboronation rates to be highly dependent on the relative positions of the boron and heteroatom substituents, sometimes in surprising ways (**4** to **8**, Figure 1B). For example, 5-pyrazolylboronic acid (**6**) exhibits a relatively simple profile (pathways ii and iv), whereas the regioisomer (**7**) has a much more nuanced one (pathways i, ii, iv, and v). DFT identified several key interactions that assist boronate departure in the protodeboronation transition states. For example, hydrogen bonding assists B(OH)_3_ departure for 2-pyridyl boronic acid (**8**), Figure 2, leading to the highest reactivity at neutral pH, where the species is zwitterionic (**5**_H,OH_, via pathway iv). This interaction is absent in the 3-pyridyl isomer (**8**), leading to much greater stability. In the 5-thiazolyl system (**4**), the σ*_C–S_ orbitals assist B(OH)_3_ departure, and the addition of *N*-coordinating metal salts, *e.g.* ZnCl_2_, enhances this, leading to rate acceleration. The opposite effect is observed with 2-pyridyl boronic acid (**6**) where metal salts block the H-bonding.^1^

### 2.5 Boronic Esters

**Figure 3:**
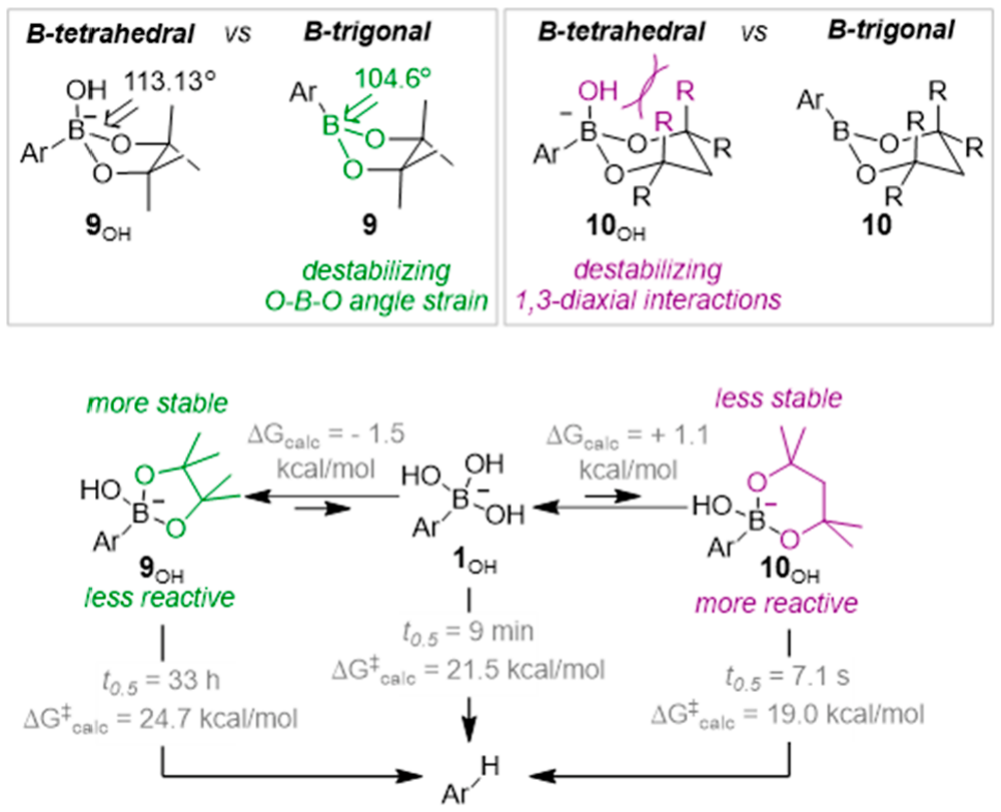
Contrasting effects of ring size on stability of esters (**9**/**10**) and their hydroxyboronate anions (**9**_OH_/**10**_OH_). Ar = 2,6-difluorophenyl. Data from ref (2).

Use of a boronic ester rather than the acid can provide increased shelf life, ease of manipulation/purification, and stability toward protodeboronation under basic cross-coupling conditions.^13,14,16^ Prime examples of this are the ubiquitous pinacol boronic esters, *e.g.*, **9** (Figure 3). However, in a recent *in situ*^19^F NMR investigation we showed that this stabilization is not general, with some classes of ester undergoing substantially accelerated protodeboronation.

**Figure 4:**
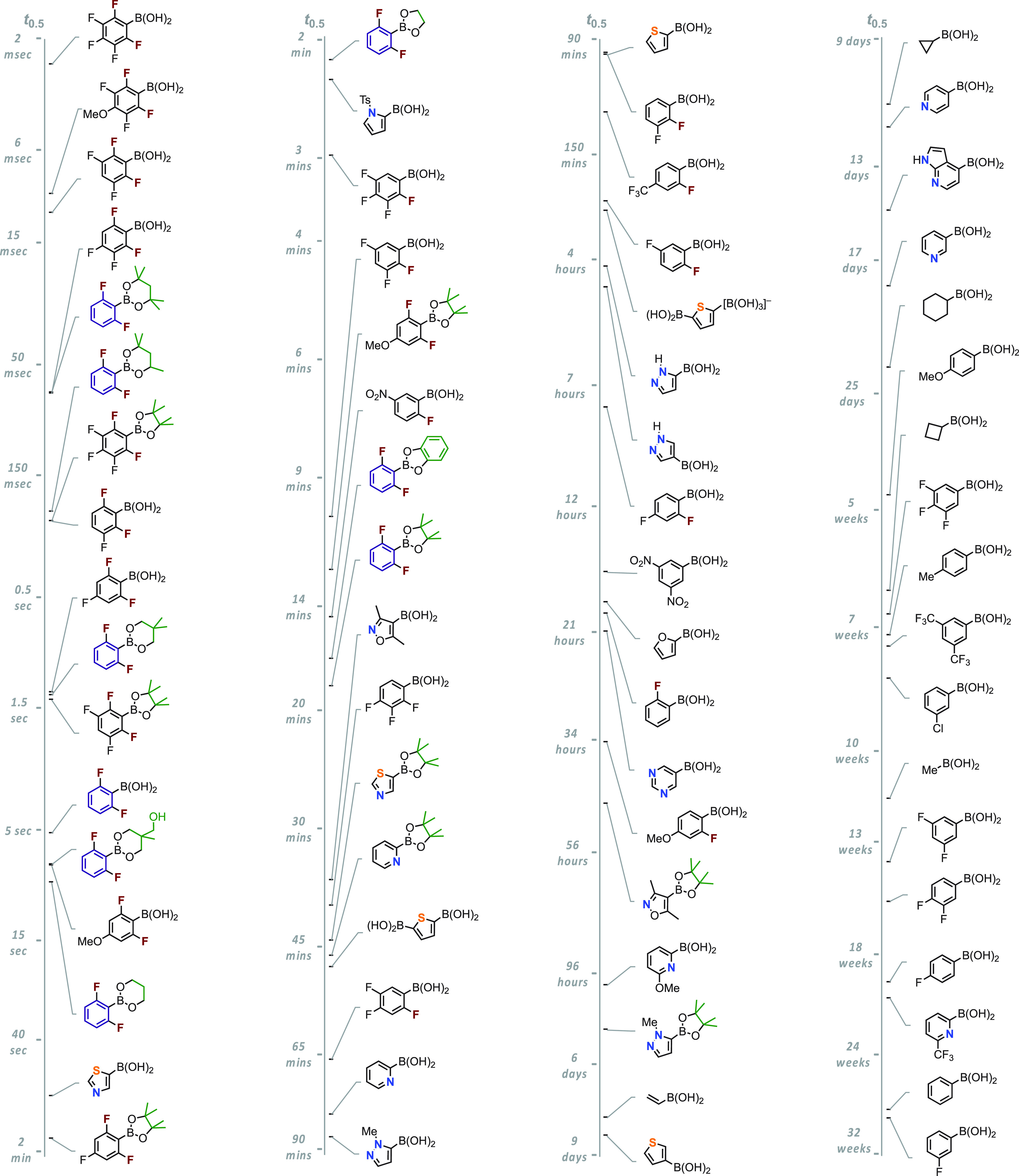
Comparison of the protodeboronation rates of hydroxyboronate anions generated from the corresponding boronic acid or ester at pH 13, in 50% aq. dioxane at 70 °C; some structural features are highlighted in color to aid comparison. Vertical axes indicate approximate half-lives (log_10_-scale). Half-lives for the ester are for *direct* protodeboronation (*k*_BE_) only, see Figure 1D, and have been extrapolated by reference to their rate-ratio with the corresponding boronic acid at 21 °C. The half-lives at 21 °C can generally be estimated using *t*_0.5_ ≈ 10^(2.15+1.13log{*t*})^ where {*t*} is the half-life at 70 °C, in seconds. Data from refs (1), (2), and (30).

DFT calculations showed that the acceleration arises when there is significant steric strain in the tetrahedral boronate that is generated on addition of the hydroxide ion to the trigonal boron center of the ester.^2^ This is typically found in esters generated from highly alkylated 1,3-propanediols, in other words, those that lead to 1,3-diaxial ring strain in the cyclic boronate (*e.g.*, **10**_OH_, Figure 3). These can undergo base-mediated protodeboronation 2 orders of magnitude more quickly than the corresponding boronic acid. Conversely, considerably less strain is present in the tetrahedral boronates generated from five-membered ring esters, *e.g.*, **9**_OH_,^31,32^ resulting in enhanced stability and genuine “protection”.^2^ The range of stability of the esters can be compared with boronic acids in Figure 4, where they are arranged in order of half-lives of the hydroxyboronate anions at 70 °C.

However, the situation is not as simple as the generalizations in Figure 3 suggest. A key issue is that the aqueous organic medium that induces direct protodeboronation (*k*_BE_, Figure 1D) also mediates ester hydrolysis,^2,33^ resulting in a competing indirect “prehydrolytic” route (*k*_hyd_, *k*_BA_) via the trihydroxyboronate.^2^ This “leakage” has the effect of reducing the effective stabilization by the five-membered ring esters. For example, at high pH the pinacol ester-ate complex **9**_OH_ undergoes about 70% indirect protodeboronation, even though the trihydroxyboronate (**1**_OH_) does not significantly accumulate (≤1%, see inset in Figure 1D).^2^ Computed barriers for boronic ester protodeboronation indicated concerted fragmentation–protonation and direct fragmentation mechanisms, analogous to I and II, Figure 1C.^2^ Although the lowest-energy pathways correlated well with observed rates, with the more reactive examples, typically electron-deficient aromatics, proceeding via pathway II, the absolute barriers were anomalously low. This triggered our development of an improved computational protocol for the systematic placement of solvent molecules for specific solvation of the boronates.^2^

## 3 The Ruppert–Prakash Reagent, TMSCF_3_

TMSCF_3_ was introduced in 1984 by Ruppert as a *de novo* CF_3_-source,^7^ and its use in organic synthesis was pioneered soon after by Prakash.^8^ It is now a core reagent in the synthesis of fluorochemicals, available at scale, easy to handle, relatively cheap, and the starting material for many other CF_3_-transfer reagents.^9,10^ Recent advances by Prakash and Hu have greatly expanded the application of TMSCF_3_ as a versatile CF_2_-source,^34−36^*e.g.*, for generation of difluorocyclopropa(e)nes,^34,37^ TFE,^35^ perfluoroalkylmetallics,^36^ and other difluoromethylenes.^38^ In all applications, the reagent is used in excess, typically 2–5 equiv.

**Scheme 2:**
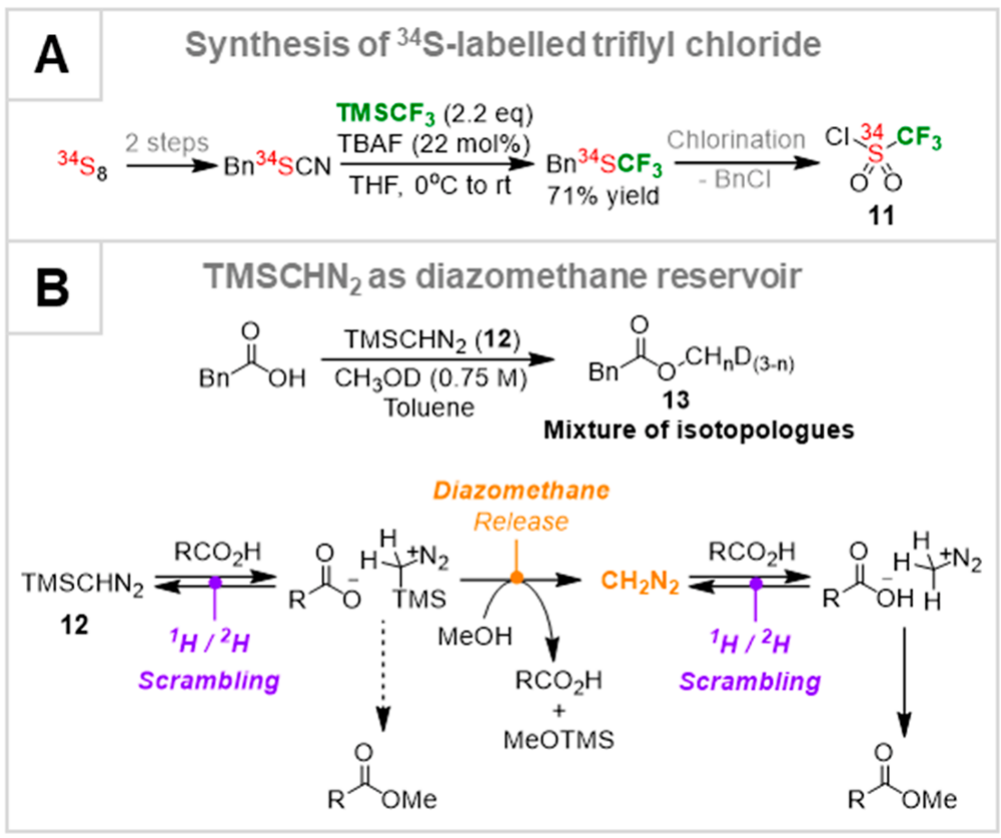
(A) TMSCF_3_ in the Synthesis of ^34^S-11; (B) Mechanism of Methyl-Esterification by TMS-diazomethane (12)

In 2008 we needed to prepare ^34^S-triflyl chloride (**11**), for a mechanistic study of the anionic thia-Fries rearrangement.^39^ After considerable exploration of other methods, we developed a route from ^34^S_8_, in a sequence involving delivery of CF_3_ from TMSCF_3_, Scheme 2A.^39,40^ At about the same time we required various ^2^H-labeled methyl esters for a study of homoallylcyclopropanation.^41^ Given the accepted mechanism for Aoyama–Shiori methylesterification with TMS-diazomethane (**12**, Scheme 2B),^42^ replacing MeOH by MeOD should have given monodeutero esters. Instead, we obtained all four isotopologues, RCO_2_CH_*n*_D_(3–*n*)_, (**13**). After detailed investigation, we elucidated that CH_2_N_2_ is generated transiently *in situ*,^43^ which is another example of a benefit of “slow-release”. These investigations led us to develop an interest in the role of anions as initiators for nucleophilic transfer of organic fragments from organosilanes to electrophiles, including from Ar-TMS to Au,^44^ and eventually to detailed studies of TMSCF_3_.^3,4,45^

### 3.1 Siliconates and the Trifluoromethanide Anion

**Scheme 3:**
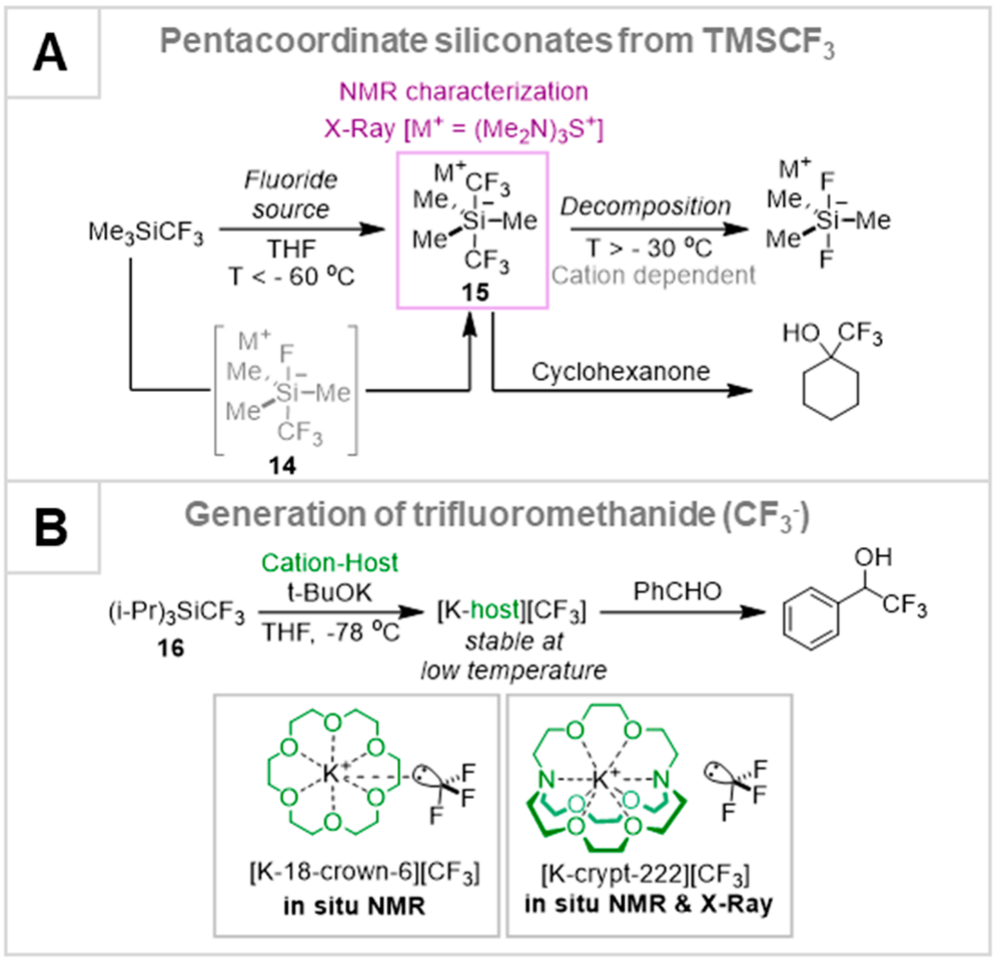
(A) Anion Addition to TMSCF_3_ to Generate Thermally Labile Siliconates 14 and 15; (B) Generation of Trifluoromethanide ([CF_3_]^−^[ML]^+^) from Bulky Silane 16

The stoichiometric reaction of TMSCF_3_ with nucleophilic anions had already been studied experimentally, and in considerable detail.^46−50^ The two principal findings are summarized in Scheme 3. In 1999, Naumann^46^ and Kolomeitsev and Röschenthaler^47^ independently showed that addition of a silaphilic anion to TMSCF_3_ generates pentacoordinate siliconates **14** and **15** that rapidly decomposed above −30 °C. Most, but not all, interpretations of anion-mediated reactions of TMSCF_3_ invoke *direct* transfer of CF_3_ from a siliconate (*e.g.*, **14** or **15**) to the electrophile, Scheme 3A.^10^ About 15 years later, Prakash^48^ and Grushin^49^ independently showed that trifluoromethanide ([CF_3_]^−^) could be generated at low temperatures from bulky silane **16**, by using *t*-BuOK with a crown ether^48^ or a cryptand.^49^ The free carbanion ([CF_3_]^−^) was even characterized by cryogenic single-crystal X-ray diffraction.^49c^ In all cases, addition of electrophiles such as ketones and aldehydes to the reaction mixtures at low temperature generated the corresponding CF_3_-addition products.^46−48,49a^

**Figure 5:**
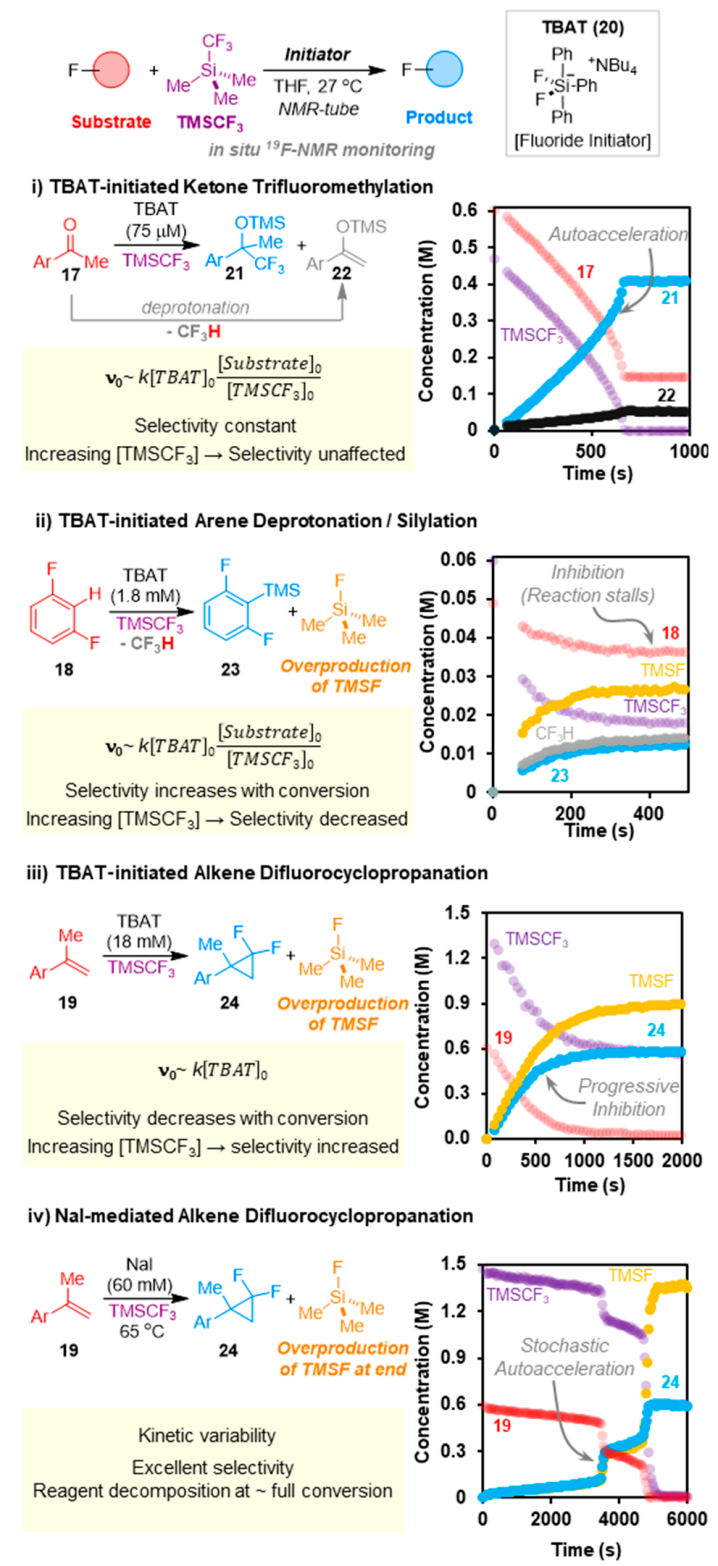
Examples of *in situ*^19^F NMR reaction profiles for the reactions of TMSCF_3_ with ketone (**17**), arene (**18**), and alkene (**19**), together with factors affecting selectivity and the initial rate (*v*_0_) of TMSCF_3_-consumption. Data from refs (3), (4), and (45).

These prior analyses provided us with a framework to interpret the kinetics and mechanism of CF_3_ transfer, and later also CF_2_, from TMSCF_3_ after initiation with substoichiometric anion at ambient temperature. We focused on the addition of CF_3_ to *p*-F-acetophenone (**17**),^3^ Kondo silylation of 1,3-difluorobenzene (**18**),^4,51^ and the difluorocyclopropanation of *p*-F-α-methylstryene (**19**)^4^ (Figure 5). Intriguingly, all three processes can be conducted using the same anhydrous fluoride-based initiator (“TBAT”; **20**)^52^ in THF at ambient temperature. This feature allowed us to interrogate how the substrate, the only variable, affects the behavior of the system. After careful adjustment of concentrations, and use of high-purity TMSCF_3_,^3^ all three reactions (Figure 5i–iii) were amenable to detailed *in situ* analysis by ^19^F NMR spectroscopy.^3,4,12,45^

### 3.2 Fluoride-Initiated CF_3_ Transfer

Differences in behavior between the three reaction classes, in terms of both initial rates (*ν*_0_) and selectivities, are evident in Figure 5i–iii. The addition of CF_3_ to ketone **17** proceeds with autoacceleration when [**17**]_0_ > [TMSCF_3_]_0_, as in the example shown in Figure 4i, where the product (**21**) curve has a rising gradient. Conversely, when [**17**]_0_ < [TMSCF_3_]_0_, the reactions become progressively slower. The only major side reaction involves the *O*-silylation (**22**) of enolizable ketones. This cogenerates CF_3_H and proceeds throughout the reaction in a constant proportion relative to the addition.^3^

The Kondo silylation^51^ of arene **18** displays kinetics analogous to the ketone, but in the example shown in Figure 4ii, [**18**]_0_ < [TMSCF_3_]_0_ and the reaction becomes progressively slower, eventually stalling.^45^ Moreover, a major side reaction, not involving arene **18**, converts TMSCF_3_ into TMSF and a range of perfluoroalkenes, *vide infra*. The kinetics of the difluorocyclopropanation of **19** (Figure 5iii) are very distinct from the other two cases, with the initial rate of TMSF generation independent of both [**19**]_0_ and [TMSCF_3_]_0_.^4^ The major side reaction is the overproduction of TMSF. This is also found for the NaI-mediated process (Figure 5iv)—but only in the final phases of reaction.^4^

**Figure 6:**
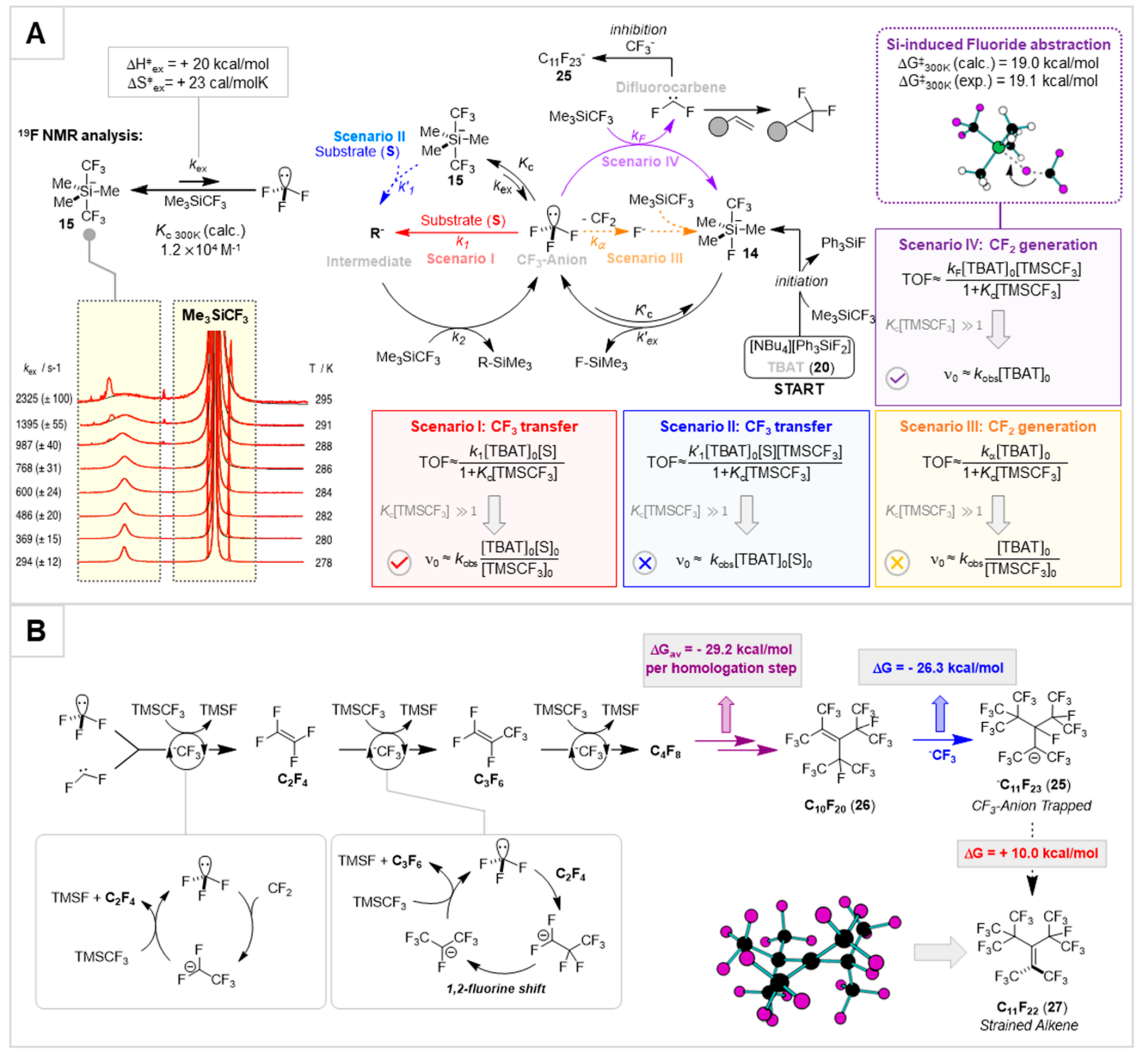
(A) Overarching mechanism highlighting the kinetics and mechanism of CF_3_ and CF_2_ transfer from TMSCF_3_, after initiation by substoichiometric TBAT (**20**). The ticks and crosses indicate which mechanistic scenarios (I, II, III, IV) are consistent with the experimentally determined kinetics. S = substrate, *e.g.***17** or **18**; R-SiMe_3_ = product, *e.g.***21** or **23**. (B) Pathways for TMSCF_3_ decomposition leading to anion-sequestration and inhibition. Data from ref (4).

In all three of the TBAT-initiated reactions (Figure 5i-iii), the *in situ*^19^F NMR signal of the TMSCF_3_ at ambient temperature exhibits dynamic line-broadening.^3,4,45^ At lower temperatures, the siliconate **15** is detected, and variable temperature line-shape analysis (Figure 5A) allowed extraction of the rate of CF_3_-decomplexation (*k*_ex_; Δ*H*^‡^ = 20 kcal/mol; Δ*S*^‡^ = 23 cal/mol K). Although DFT calculations indicate the equilibrium very strongly favors **15** over free [CF_3_]^−^, the rapidly reversible decomplexation (*k*_ex_; Δ*G*^‡^ = 13.1 kcal/mol at 27 °C) leads to fast exchange of CF_3_-groups between TMSCF_3_ and **15** and dynamic line-broadening in both. In terms of the productive reactions (Figure 5i,ii), siliconate **15** is either a passive anionic reservoir (scenario I, Figure 6A) or directly transfers the CF_3_ to the substrate (scenario II).^10^ Analysis of the kinetics allowed dissection of this dichotomy: only in scenario I can the TMSCF_3_ inhibit the rate of CF_3_-transfer to the substrate. It does this by sequestering (*K*_c_) the [CF_3_]^−^, thus attenuating the rate of the anionic chain reaction. DFT-calculations strongly supported these findings by showing that *direct* anionic CF_3_-transfer from the siliconate **15** (scenario II) to *any* electrophile or acid involves a prohibitive (>50 kcal/mol) umbrella-like CF_3_-inversion. Instead, transfers must proceed via predissociation of the [CF_3_]^−^.^3^ Bulkier R_3_SiCF_3_ reagents have a lower affinity (*K*_c_) for [CF_3_]^−^ and lead to more efficient CF_3_-transfer (R = Et) or to a change in rate-limiting step (R = iPr).^3^

### 3.3 Fluoride-Initiated CF_2_ Transfer

A variety of tests, including heavy-atom KIEs,^53^ relative reactivity of *E*/*Z*-alkenes, and linear free-energy relationships, confirmed that the difluorocyclopropanation reactions proceed via concerted capture of singlet difluorocarbene, CF_2_ (**19** → **24**, Figure 5iii,iv).^4^ The consensus in the literature was that the CF_2_ is generated via spontaneous α-fluoride elimination from [CF_3_]^−^ (scenario III, Figure 6A).^54^ However, the kinetics under fluoride initiation were not at all consistent with this, because TMSCF_3_ should inhibit the reaction (*K*_c_), via generation of siliconate **15** (Figure 6A, scenario III). We thus computationally explored the direct extrusion of CF_2_ from siliconate **15**; however, all attempts to locate a transition state for this diverted to an *intermolecular* fluoride transfer from C to Si, scenario IV. Detailed stopped-flow ^19^F NMR spectroscopic analyses of the difluorocyclopropanation of **19** between 2 and 18 °C gave activation parameters and kinetics fully consistent with scenario IV,^4^ with fluoride-abstraction (*k*_F_) occurring approximately once in every 10^5^ reassociations (*K*_c_) of [CF_3_]^−^ with TMSCF_3_.

### 3.4 Perfluoroalkenes and Inhibition of the Chain Reaction

During *in situ*^19^F NMR spectroscopic analysis of the Kondo silylation of **18** and the difluorocyclopropanation of **19** (Figure 5), numerous low-intensity complex multiplets appear in the ^19^F NMR spectra.^4,45^

This phenomenon was kinetically linked with progressive inhibition. Both reactions proceed by anionic chain reactions (Figure 6A, scenarios I and IV), and thus, inhibition involves diversion of the active chain-carrier(s) into inert, *i.e.*, nonsilaphilic, anions. The major component of these was identified as the known perfluoroalkyl anion [C_11_F_23_]^−^ (**25**),^50^ albeit with a structure revised on the basis of ^19^F–^19^F NOESY and ^n^*J*_FF_ values.^45^

Computational investigation of the thermodynamics of sequential CF_3_-addition, 1,2-fluorine shifts, and fluoride-elimination^55^ allowed us to understand why a C_*n*_F_2*n*_/[C_*n*_F_2*n*+1_]^−^ cascade leads to, and ceases at, C_11_, *i.e.*, **25** (Figure 6B). Each alkene homologation step is favorable (Δ*G*_av_ = −29.2 kcal/mol) until [CF_3_]^−^ adds to C_10_F_20_ (**26**) to generate anion **25**. At this point, fluoride-elimination becomes disfavored (Δ*G* = +10.0 kcal/mol) because of the steric strain in the resulting alkene, C_11_F_22_ (**27**).^4^ In other words, anion **25** acts as a thermodynamic “sink”, trapping [CF_3_]^−^ and F^–^ and terminating the desired anionic chain reactions.^4,45^

### 3.5 Productive Fractionation, *f*

**Figure 7:**
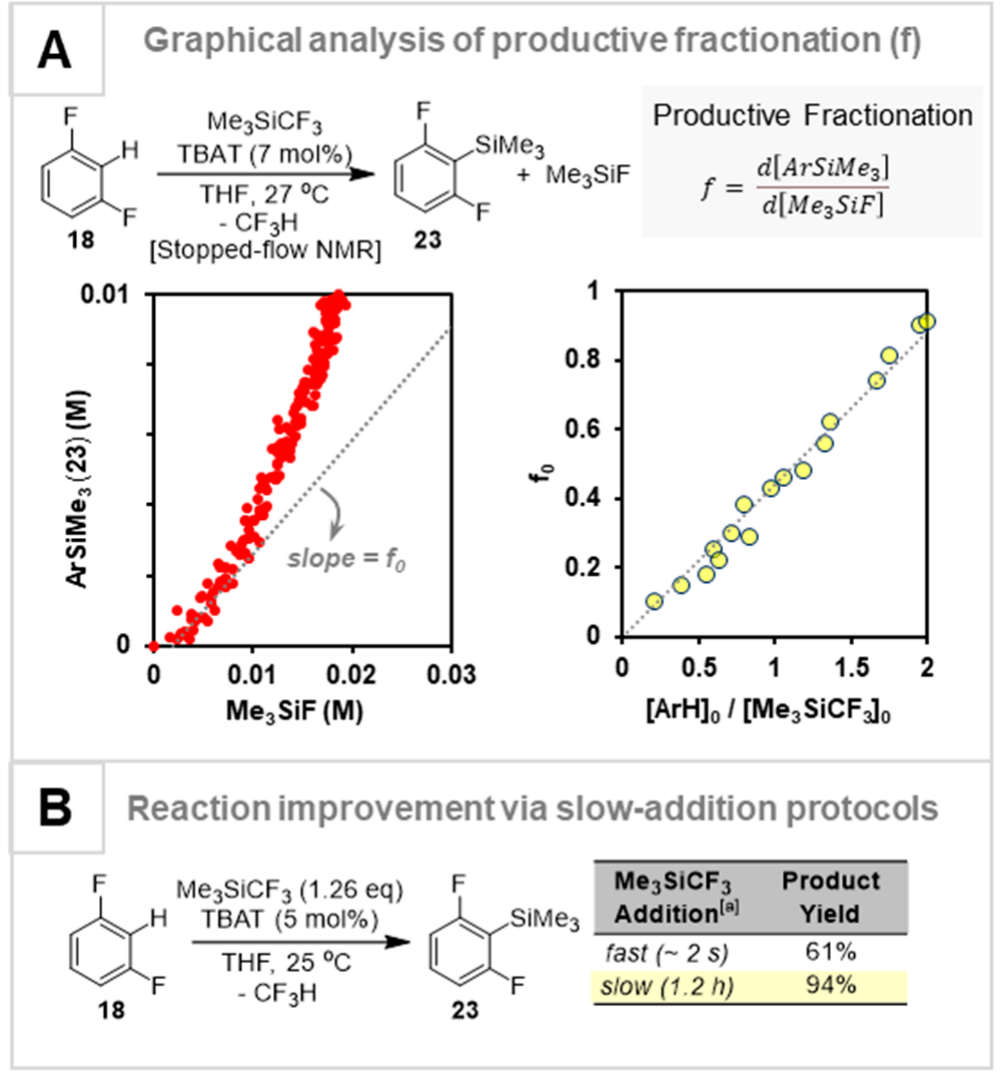
(A) Analysis of the productive fractionation, *f*, of TMSCF_3_ into desired (Ar-TMS, **23**) and undesired (TMSF) products during silylation of **18**. The fractionation increases as the reactions proceed and as the initial ratio [**18**]_0_/[TMSCF_3_]_0_ is raised. (B) Insight from the changes in *f*, informing the slow addition of TMSCF_3_. Data from ref (45).

TMSF evolution acts as reporter for the net loss of CF_2_ from TMSCF_3_. This can be used to quantify the extent of side-reactions versus product in the form of a productive fractionation parameter: *f* = d[Product]/d[TMSF].^4,45^ Graphical analysis of *f*, Figure 7A, allows assessment of how the changes in concentration of the various reaction components, within or between reactions, affects the efficiency. These analyses proved fruitful: by deliberately keeping a low concentration of the “problematic” component, the productive fractionations can be enhanced. For example, the Kondo silylation could be improved to near-quantitative conversion of **18** to **23** by slow addition^45,56^ of TMSCF_3_, Figure 7B.

### 3.6 NaI-Mediated CF_2_ Transfer

**Scheme 4:**
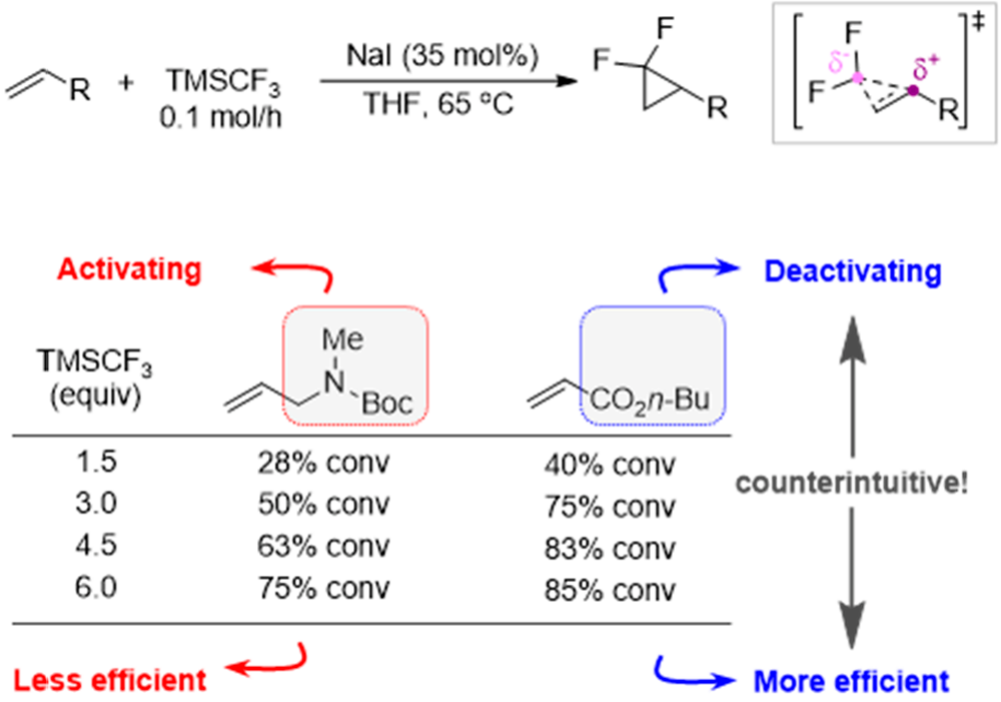
Counterintuitive Results with Alkenes That Are Deactivated Toward CF_2_-Cycloaddition Data from ref (56).

The most effective synthetic method for alkene difluorocyclopropanation with TMSCF_3_ employs NaI, a process pioneered by Prakash and Hu.^34^ The conditions afford substantially enhanced substrate scope, including alkynes, and are also effective for *in situ* generation of tetrafluoroethylene.^35,36^ Grygorenko^56^ has shown that slow addition of TMSCF_3_ and NaI allows efficient difluorocyclopropanation of alkenes which are considered “unactivated” toward CF_2_ cycloaddition (Scheme 4), considerably expanding the scope of application.^37^

Mechanistically, the NaI-mediated reactions of TMSCF_3_ proved highly vexing, with many initially counterintuitive features. The reactions are characterized by transient and apparently stochastic autoaccelerations;^4^ indeed, this exothermic process can proceed violently upon scale-up.^37a,56^ During this quasi-stochastic phase (see Figure 5iv), the productive fractionations are excellent (*f*_0_ > 0.99) with very little overproduction of TMSF, irrespective of the concentrations of any of the reaction components. However, at some point, and in an unpredictable manner, the rate of TMSF generation surges and *f* drops precipitously. Alkene **19** undergoes quantitative difluorocyclopropanation, and the majority of the excess TMSCF_3_ is converted into a broad range of fluorocarbons, *vide infra*.

This distinctive reactivity requires the presence of both the sodium and the iodide, and despite much effort, the primary initiation of these processes remains unclear.^61^ The NaI concentration does not affect the initiation rate, and DFT calculations indicate prohibitively high energies for all direct reaction pathways with TMSCF_3_. Overall we concluded that initiation must be “effected by traces of unidentified silaphilic species generated in situ from the NaI, by oxidation, reaction with decomposition products of the TMSCF_3_, or coreaction with the Lewis basic THF solvent, or by species already present in the NaI from the supplier.”^4^ What was clear is that suitably reactive carbonyls, *e.g.*, *p*-F-benzaldehyde (**28**), undergo addition of both [CF_3_]^−^ and, in trace quantities, [CF_2_I]^−^.^57^ However, there were none of the characteristic signals^3,4,45−47^ for siliconate equilibria (*K*_c_) evident in the *in situ*^19^F NMR analyses, even at low temperatures.

**Figure 8:**
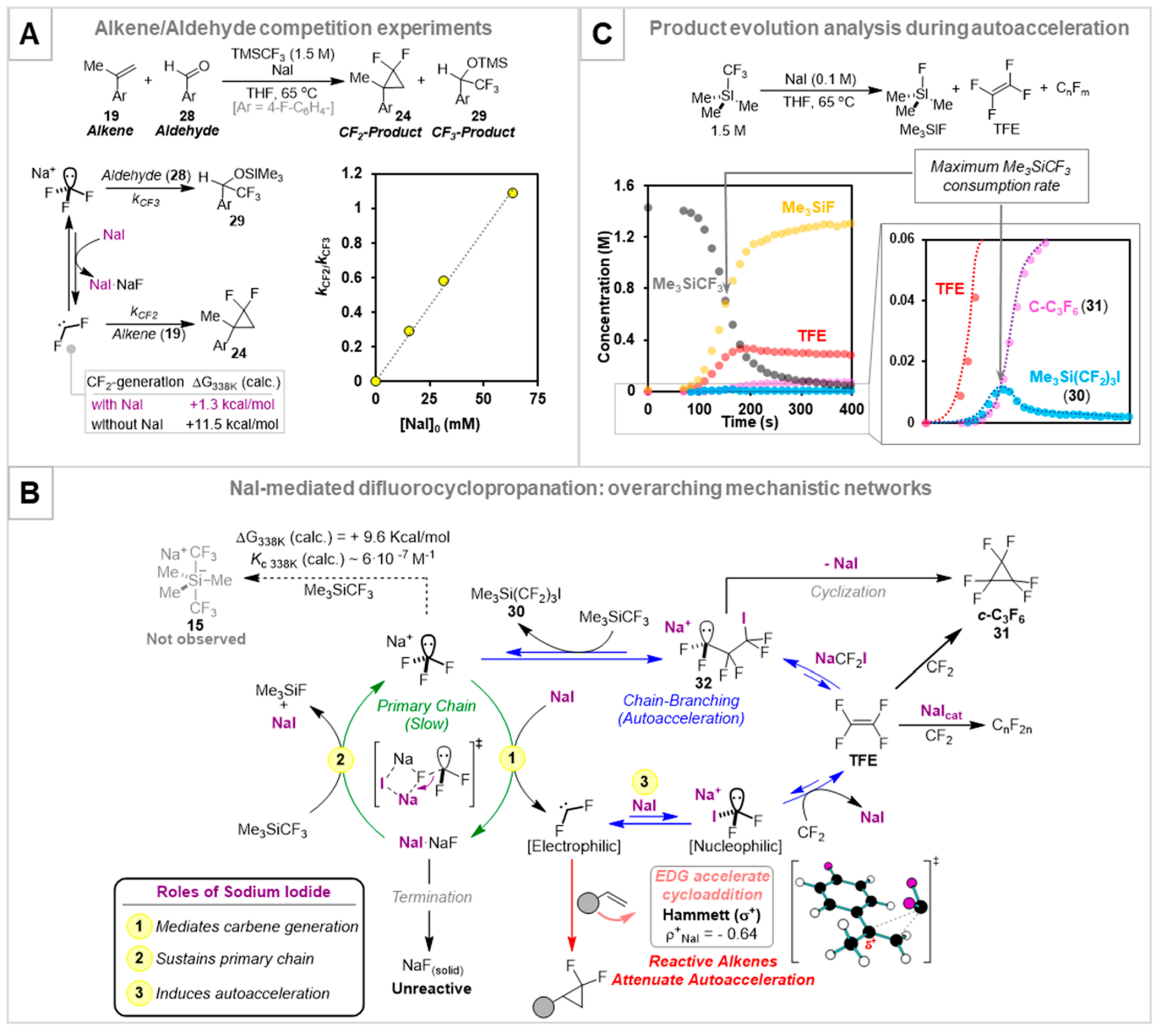
(A) Partitioning analysis by competition of alkene **19** with aldehyde **28** during NaI-mediated difluorocyclopropanation. (B) Chain reaction identified for CF_2_-generation from TMSCF_3_. (C) NaI-mediated decomposition of TMSCF_3_ in the absence of alkene **19**, and identification of species arising from chain-branching autoacceleration. Data from ref (4).

The timing and magnitude of the transient autoaccelerations varied greatly from run to run making standard time-based kinetic analyses very unreproducible. We thus tackled the problem by competition experiments, using fractional conversion of substrates as a time-independent parameter to characterize the various processes involved. For example, coreaction of alkene **19** with aldehyde **28** provided **24** and **29** as an indirect measurement of the CF_2_ and [CF_3_]^−^ present in the reaction, in the form of a first-order partitioning factor (*k*_CF_2__/*k*_CF_3__). The linear relationship between *k*_CF_2__/*k*_CF_3__ and [NaI]_0_, Figure 8A, indicated that CF_2_-generation from [CF_3_] ^–^ involves NaI, and DFT studies suggested an assisted α-fluoride elimination and stabilization^58^ of the nascent NaF (see “primary chain” in Figure 8B).

Structures that see association of ions in solution, and that can undergo dynamic exchange of partners, present another challenge for computation. We informally described our initial attempts to understand the counterion effects on the speciation in Figure 8A,B as “*molecular paintball*”: exotically colored spheres representing the cations were placed around the relevant anions, relying on intuition. The protocol that was first applied for solvation of the boronate species^2^ (*vide supra*) is now being extended to placement of counterions, this being driven by a need to be able to approach such situations more logically in the future.

Identifying the origins of the autoacceleration was challenging, not least because the onset and duration is very nonpredictable.^4,58^ The surge in consumption of the TMSCF_3_ when the alkene is depleted suggested that CF_2_-accumulation triggers the autoacceleration. Consistent with this, reactions conducted without alkene **19** present enter autoacceleration after a short but variable induction period.^4^ Detailed ^19^F NMR spectroscopic analysis of the temporal evolution at high NaI concentrations proved informative (Figure 8C). At first, TFE is generated, followed by an intermediate tentatively identified as TMSCF_2_CF_2_CF_2_I (**30**)^4^ and then perfluorocyclopropane (**31**), with a clear correlation: the temporal concentration of [**30**] mirrors the rate of consumption of the TMSCF_3_. Taken together, the observations suggested that transient carbanionoid [Na][(CF_2_)_3_I] (**32**) induces “chain-branching” (Figure 8B), a classic origin of rapidly accelerating reactions.

The requirement for TFE and CF_2_ accumulation to indirectly induce chain-branching explains several initially confusing or counterintuitive results. For example, alkenes that are activated toward CF_2_ dampen the autoacceleration, and thus, reactions employing less activated alkenes and alkynes can lead to faster overall difluorocyclopropanation, Scheme 4.^4,56^ Also, in contrast to fluoride initiation, most alkynes undergo selective reaction under the NaI conditions, without competing double-addition of CF_2_. This is because the TFE that accumulates in the autoacceleration phase has a low barrier to CF_2_ cycloaddition (Δ*G*_338_^‡^ = 12 kcal mol^–1^), allowing bypass of excess CF_2_ into *c*-C_3_F_6_ (**31**) and other perfluorocarbons, Figure 8B, rather than consuming the desired difluorocyclopropene product. The mechanistic features also provide an explanation for the greatly improved efficiency under Grygorenko’s conditions.^56^ Slow addition of the TMSCF_3_/NaI gives time for the endogenous TFE to dissipate or decay, delaying or attenuating intense autoacceleration and maintaining a high productive fractionation, *f*.

## 4 Conclusion

This Account has summarized some of our mechanistic work on popular classes of organoboron^1,2,24,25,30^ and organosilicon^3,4,45^ reagents. A recurring theme to the investigations has been the use of kinetics, NMR, isotope-effects, and partitioning analysis, *i.e.*, measuring the selectivity of a process as a function of conversion or reactant concentration. Partitioning analysis has the benefit of removing the time-dependency component for reactions that cannot easily be controlled or that proceed with unpredictable rates.

All of the studies benefitted from a deeply collaborative combination of experiment and computation. Crucially, this was initiated at the very beginning of each investigation and in two separate research groups. This facilitated the development, testing, and revision or elimination of a large variety of hypotheses. Indeed, were it not for this two-centered collaborative arrangement that enforced discussion, reflection, more-rigorous logic, and the tensioning of experiment and theory, we would not have elucidated many of the features outlined above. The collaborative process also highlighted gaps in both experimental and computational methodologies that we then sought to fill.^2,3,11^

Both areas of investigation were initiated after making unexpected observations in unrelated projects. Indeed, the work has been almost entirely curiosity-driven without predefined goals. It has nonetheless yielded insights that are of considerable practical utility, an outcome inconceivable to some research funding administrators. For example, investigation of the mechanism of hydrolysis of trifluoroborate salts led us to develop nonetching conditions for their synthesis,^24c^ a process that has now been optimized and applied industrially at >10 kg scale.^59^ Insights into the hydrolytic processes involved in C–O and B–N cleavage in MIDA boronates^25^ aided Burke in the design of a new class of hydrolysis-resistant TIDA boronates,^60^ widely expanding the scope of application, and also led us to develop new parameters for nucleofugality at boron.^61^ By study of over 70 different boronic acids and esters we have shown that their protodeboronation can be defined by six different pathways, modulated by pH speciation; concentration (self/autocatalysis); and their (hetero)aromatic, alkyl, or vinyl structure.^1,2,30^ The protodeboronation half-lives at high pH span nearly 10 orders of magnitude, Figure 4. Analogously, the anionic chain reactions that lead to decomposition of TMSCF_3_ have been identified and kinetically delineated.^3,4,45^ This allows the mechanism-informed design of conditions for maximizing the productive fractionation of the TMSCF_3_ into the desired CF_3_- or CF_2_-derived product and for the safer scale-up of these processes.^37,56^
